# Cholera resurges in Zambia: Challenges and future directions

**DOI:** 10.1016/j.ijregi.2025.100640

**Published:** 2025-03-28

**Authors:** Clyde Moono Hakayuwa, Olivier Sibomana, Chapline Shike Kalasa

**Affiliations:** 1 Department of Public Health, Copperbelt University, Ndola, Zambia; 2 Department of General Medicine and Surgery, College of Medicine and Health Sciences, University of Rwanda, Kigali, Rwanda; 3 Department of Public Health, Tropical Diseases Research Centre (TDRC), Ndola, Zambia

**Keywords:** Cholera outbreaks, Water, sanitation, and hygiene (WASH), Health care infrastructure, Epidemic preparedness, AI-driven surveillance

## Abstract

Cholera remains a major global health challenge, disproportionately affecting low-income countries with inadequate water, sanitation, and hygiene (WASH) infrastructure. Zambia, historically prone to outbreaks, experienced a severe resurgence in 2023-2024, with over 10,887 cases and 432 deaths. The contributing factors include poor WASH access, systemic health care weaknesses, stigma, and logistical barriers. Only 32% of households have basic water services, and health care worker shortages persist, especially in densely populated areas. Misinformation and cultural practices hinder public health efforts, whereas fiscal constraints limit prevention strategies. Strengthening epidemic preparedness through WASH investments, systems thinking, predictive modeling, artificial intelligence–driven surveillance, and multi-sectoral approach integrating community engagement and health system resilience is essential to mitigate the outbreak. This commentary explores Zambia's cholera control challenges and outlines strategic directions for a resilient, sustainable response to this enduring public health threat.

## Highlights

• Gaps in water, sanitation, and hygiene infrastructure contribute to recurring cholera outbreaks.
• Health care weaknesses—supply shortages, staffing, logistics—hinder outbreak response.
• Misinformation, stigma, and culture fuel cholera spread and delay interventions.
• Shifting from reactive to proactive epidemic preparedness is crucial.
• A multi-sectoral approach—community, modeling, resilience—mitigates outbreaks.

## Introduction

Cholera is a waterborne disease caused by the toxigenic bacterium *Vibrio cholerae* strains from serogroups O1 or O139, spreading primarily through the fecal-oral route [1]. It has plagued human populations for centuries, with descriptions dating back to the 5th century BC [2]. Its modern understanding began in the 19th century with John Snow's pioneering work on its transmission via contaminated water, which laid the foundation for modern epidemiology [2]. Cholera remains a global public health challenge, disproportionately affecting low-income countries where inadequate water, sanitation, and hygiene (WASH) infrastructure persist. Annually, the World Health Organization (WHO) estimates 1.3-4.0 million cholera cases and 21,000-143,000 deaths worldwide. However, many cases go unreported because of limited surveillance and fears of economic repercussions on trade and tourism [3].

Africa bears a significant burden of the disease, accounting for over half of global cholera cases reported in recent years [4]. Zambia is counted among many sub-Saharan African nations that remains vulnerable because of recurrent WASH challenges. Historical data show that Zambia experienced 29 cholera outbreaks between 1977 and 2018, with case fatality rates ranging from 0.5% to 9.3%. Major outbreaks, such as those in 1991, 1999, 2010, and 2018, highlighted the cyclical nature of the disease in the country during the rainy season [5].

The current resurgence of cholera in Zambia began in October 2023, with Lusaka Province as the epicenter. By January 2024, over 10,887 cases and 432 deaths were reported across nine provinces, with Lusaka, Central, and Eastern provinces being the hardest hit [6]. Notably, the case fatality rate (4%) during this outbreak exceeded the WHO threshold, underscoring the severity of the crisis. Challenges in the vaccination campaign, exacerbated by global vaccine shortages, further compounded the situation. Despite these difficulties, the government-initiated measures such as distributing clean water, establishing oral rehydration points, and opening cholera treatment centers, the largest being at the National Heroes Stadium in Lusaka [7].

The resurgence of cholera in Zambia represents not only a public health concern but also a socio-economic and developmental challenge. It undermines the objectives of the Zambia Multisectoral Cholera Elimination Plan and Global Task Force on Cholera Control, which aims to reduce cholera-related deaths by 90% by 2025 and 2030, respectively [8,9]. Recurring outbreaks place immense strain on health care systems, disrupt education through school closures, and weaken the economy by lowering productivity and diverting critical resources to emergency response effort. The 2024 outbreak, the most severe in two decades, sharply highlighted the fragility of Zambia's health infrastructure and the urgent need for systemic improvements to build resilience against future epidemics [6].

In 2025, the Ministry of Health reported new cholera cases, indicating that the outbreak persists. As of January 2, 2025, 15 cases were recorded in Nakonde—a major trading hub in northern Zambia—marking its first outbreak [[8], [10]]. By January 30, 2025, the outbreak had spread to other parts of the country, with 70 cases recorded in Chililabombwe, 21 in Nakonde, four in Kitwe, and one in Chingola [10], [11]. This continued resurgence shows the need for sustained investments in public health infrastructure, robust surveillance systems, and community engagement to combat cholera effectively.

Although Zambia has made significant strides in combating cholera over the years, the recurrent outbreaks underscore the pressing need to address the root causes of the disease, including poverty, overcrowding, and insufficient access to safe water and sanitation. This research explores the challenges Zambia faces in controlling cholera and outlines strategic directions for a resilient and sustainable response to this enduring public health threat.

## Challenges

### Financial and logistical constraints

Zambia's financial and logistical constraints have impeded the implementation of robust cholera prevention and response strategies. Although the country is on a path to economic recovery, past fiscal challenges have left a gap in critical resources for water treatment, regular water safety testing, improved hygiene and sanitation practices, and infection prevention and control in health care settings [12].

Zambia requires approximately $5.7 billion to implement its Water Investment Programme, which aims to improve WASH infrastructure across the country. The National Urban Water Supply and Sanitation Programme has estimated a funding need of $4 billion by 2030, with a short-term requirement of $288 million in 2014. However, despite these financial needs, the 2024 national budget allocation for WASH decreased from K2.3 billion in 2023 to K2.1 billion in 2024, entailing of the persistent funding gap [[13], [14], [15]]. Although the private sector has been identified as a potential contributor to bridging this deficit, challenges such as inadequate tariffs and complex procurement processes continue to hinder investment in WASH infrastructure. These challenges should be addressed because the WHO Global Preparedness Monitoring Board [16] notes that the cost of failing to prepare for infectious disease outbreaks far exceeds the investments required to establish preventive systems. Zambia's inability to adequately finance and implement these measures could have contributed to the recurring nature of cholera outbreaks, underscoring the urgent need for a shift from reactive to proactive public health strategies.

### Lack of clean WASH facilities

The persistent lack of access to safe WASH facilities is another major driver of cholera outbreaks in Zambia. Insufficient infrastructure to provide clean water and decent sanitation has directly contributed to widespread cholera outbreaks [1].

Proactive measures in cholera prevention have been limited, leading to a recurring cycle of reactive responses that are economically and socially costly, worsening the already existing financial constraints to the country. Preventive strategies are more cost-effective than managing outbreaks [17,18], yet Zambia's approach has been predominantly reactive. According to the WHO/United Nations Children's Fund Joint Monitoring Program (2024) [19], gaps in access to essential WASH services have been noted, as shown in the Table 1.

**Table 1:** Proportion of Zambian facilities and households lacking basic water, sanitation, and hygiene services [16].

Category	Indicator	Percentage
Health care facilities	Lack basic water services	13%
Schools	Lack access to basic water	21%
	Lack basic sanitation	17%
Households	Lack basic water access	32%
Poorest quintile households	Lack access to basic water services	60%
	Lack basic sanitation	64%
	Lack hygiene facilities	82%

### Systemic weaknesses in health care infrastructure

Zambia's health care system is plagued by systemic weaknesses; there are insufficient health facilities, shortages of medical supplies, and undertrained personnel. There are only 11.2 health care workers per 10,000 people in rural areas—far below the WHO standard of 40 per 10,000—the country faces serious challenges in cholera prevention and treatment as a result. Urban areas are marginally better, with 18.7 health care workers per 10,000 people, but this still falls short of adequate coverage [20]. The shortage of health care facilities, medical personnel, and essential supplies is a widespread issue across many African countries, not just Zambia, and hinders the delivery of timely and effective health care services [21]. An adequate workforce is a fundamental prerequisite for effectively combating outbreaks. Addressing Zambia's health care workforce shortage is imperative given the WHO's warning of a global deficit of 12.9 million skilled health care professionals by 2035 [22].

The shortage of health care workers, combined with inadequate training on cholera prevention and management, has compromised the country's ability to respond effectively to outbreaks. Rural communities, which are disproportionately affected by cholera, often lack timely access to medical care because of the vast distances required to reach health care facilities.

### Misinformation, cultural practices, and stigma

Public health efforts to control cholera are undermined by misinformation, cultural practices, and stigma. The WHO recognizes misinformation and disinformation as significant threats to public health in Africa. Cultural factors, such as traditional practices (the use of untreated water from rivers and shallow wells, communal eating without proper hand hygiene, reliance on traditional healers who may use unsterilized instruments or untreated water in remedies, open defecation because of cultural beliefs about sanitation, and hesitancy to seek modern medical treatment), have also been shown to contribute to cholera transmission [23].

A study conducted in Zambia revealed that individuals diagnosed with cholera often face discrimination and social exclusion because of fear and misinformation surrounding the disease [24]. This stigma exacerbates the suffering of affected individuals and deters others from seeking timely medical intervention or participating in preventive measures.

### Urban growth, infrastructure gaps, and preparedness limitations

Lusaka, the epicenter of 75% of cholera cases in Zambia, has undergone rapid urban growth, resulting in overcrowding and inadequate sanitation infrastructure. These factors, compounded by contaminated water sources and poor sanitation practices, significantly contribute to cholera transmission among residents. Other challenges such as the inconsistent implementation of the Epidemic Preparedness and Response plan by certain District Health Officers, delays in investigating and identifying infection sources, limited experience in managing cholera outbreaks at the subnational level, high rates of community deaths, and insufficient public knowledge of cholera prevention measures further hinder efforts to eradicate the disease [12].

### Climate change

Climate change has long been a challenge for Zambia that has played a role in worsening the cholera crisis. In 2024 and 2025, the country has faced two extreme weather events: severe droughts and intense rainfall, respectively. Since February 22, 2025, heavy rains have triggered severe floods and flash floods in cholera epicenters, causing population displacement and infrastructure damage. According to the Disaster Management and Mitigation Unit, as of February 25, approximately 250 people from 50 families have been evacuated in Lusaka Province (a hotspot for cholera), many of whom are sheltering in temporary facilities [25]. Research suggests these extreme events promote cholera transmission, for example, the rising temperatures prolong the survival of cholera bacteria in water sources, whereas erratic rainfall patterns and subsequent flooding weaken already fragile sanitation systems [26]. Climate change is, therefore, another key driver of cholera outbreaks in Zambia because increasing temperatures, unpredictable rainfall, and prolonged droughts force communities to rely on contaminated water, heightening the risk of disease transmission.

**Figure 1:**
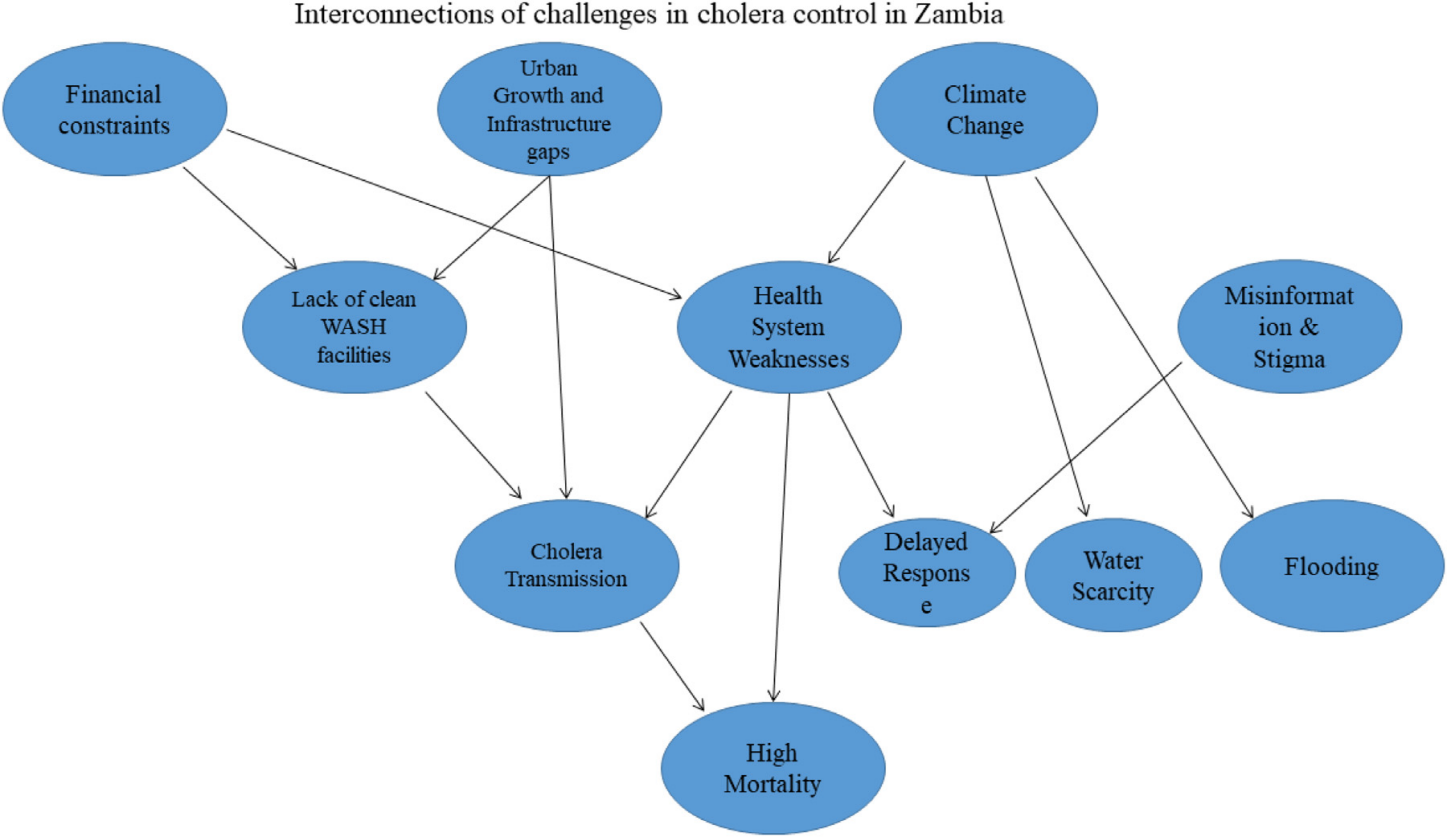
The interconnectedness of financial constraints, health care system weaknesses, urbanization, cultural factors, and climate change in driving recurring cholera outbreaks in Zambia.

These multiple challenges highlight that Zambia's persistent cholera outbreaks result from a complex interplay of financial, infrastructural, health care, and environmental factors. As illustrated in Figure 1, limited financial resources hinder investments in WASH infrastructure, leaving communities without clean water and proper sanitation, which are crucial for cholera prevention. Weak health care infrastructure and shortage of medical personnel and facilities further worsen the crisis especially in rural areas where access to timely treatment is limited. Cultural practices, misinformation, and stigma discourage individuals from seeking medical care, prolonging disease transmission. Rapid urbanization strains existing sanitation systems, whereas climate change intensifies vulnerabilities through extreme weather events that compromise water quality and sanitation infrastructure. To address these challenges there is need for an integrated, multisectoral approach that prioritizes proactive investments in prevention, health care capacity building, and climate resilience.

## Future directions

The persistent recurrence of cholera outbreaks in Zambia suggests gaps in existing response strategies that target the previously highlighted challenges. To effectively tackle this public health challenge, there is a need to strengthen and modernize our response approach by integrating innovative technologies, learning from successful interventions in other countries, and adopting more advanced, evidence-based methods. This includes enhancing surveillance systems, leveraging data-driven predictive models, and investing in sustainable WASH infrastructure to build long-term resilience against cholera outbreaks.

Strengthening surveillance and epidemic intelligence through robust systems is vital for the timely detection, notification, and response to outbreaks. Enhancing epidemiological capacity by investing in competency-based training, such as the 7-1-7 framework, equips health care professionals with the skills needed to manage outbreaks effectively at subnational levels [12].

Zambia should prioritize health emergencies in budget allocations, despite financial constraints, because these crises weaken human capital essential for economic growth. Also, investing in artificial intelligence (AI) and developing mathematical models can offer a transformative approach to addressing Zambia's seasonal cholera outbreaks. For example, in Bangladesh, predictive modeling has been used to forecast cholera dynamics, enabling targeted interventions. Through the analysis of environmental data—such as rainfall, temperature, and water quality—alongside socio-economic and epidemiologic factors, these models can help to provide early warnings that facilitate pre-emptive measures such as vaccination campaigns and improved sanitation efforts [27,28]. Implementing a similar AI-supported predictive system in Zambia could enhance preparedness, address health care deficiencies by reducing manpower needs, and improve disease response, ultimately lowering the incidence and impact of cholera outbreaks.

However, the feasibility of AI-supported solutions in Zambia must be carefully considered. Implementing AI requires solid IT infrastructure, skilled personnel, and reliable long-term datasets—resources that are currently limited within Zambia's health system. It is also noteworthy that AI-based models are only as effective as the data they rely on, and Zambia's cholera and environmental surveillance systems may not yet generate sufficiently high-quality data for accurate modeling. Strengthening surveillance should, therefore, be prioritized alongside investments in AI because this will ensure that predictive models are informed by reliable real-time data. There is also need for cost-effective assessment to determine whether AI-supported cholera prediction offers a greater return on investment compared to strengthening traditional surveillance and response systems.

Systems thinking offers a holistic framework for tackling cholera outbreaks by examining the interplay of health, environmental, and socio-economic factors. This approach helps stakeholders identify root causes and leverage cross-sectoral synergies for sustainable solutions. As shown in Figure 1, climate change, urbanization, and financial constraints are key drivers. Strengthening health financing schemes, addressing urbanization challenges, and building climate-resilient systems can enhance Zambia's ability to prevent and manage cholera outbreaks effectively. In Haiti, systems thinking was applied to integrate WASH interventions with health education and health care delivery, resulting in significant reductions in cholera cases [29]. By adopting systems thinking in Zambia, policymakers and public health officials can design strategies that address underlying determinants of cholera while fostering collaboration among government agencies, non-governmental organizations, and local communities. This integrated strategy could enhance resilience, reduce health care costs, dispel myths and misinformation through multisectoral health education, minimize the burden of seasonal outbreaks, and pave the way for long-term cholera eradication.

Investing in WASH infrastructure remains a cornerstone of cholera prevention and control because it addresses the primary drivers of the disease [12,30]. Improved access to clean water and proper sanitation facilities reduces the transmission of *Vibrio cholerae*, whereas hygiene promotion fosters behavioral changes that further limit the spread of infections. Bangladesh and Haiti have successfully minimized cholera outbreaks through sustained investments in WASH infrastructure and community health education programs. Similarly, prioritizing WASH interventions in Zambia—such as increasing investment in construction of safe water points, regular waste management, and promoting handwashing practices—can mitigate the seasonal occurrence of cholera. Scaling up WASH investments will not only prevent cholera outbreaks but also improve overall public health outcomes by reducing the burden of diseases such as typhoid fever, dysentery, and acute watery diarrhea—major public health concerns in Zambia—while contributing to Sustainable Development Goal 6 (Clean Water and Sanitation). Although initial investment costs may be high, the long-term economic and health benefits of WASH infrastructure far outweigh the recurring costs of cholera outbreak responses. Therefore, a strategic balance between immediate epidemic response and long-term WASH investments is necessary to achieve sustainable cholera control.

## Conclusion

The resurgence of cholera in Zambia calls for an urgent need for a comprehensive, multisectoral approach to address this ongoing public health challenge. Despite progress in managing outbreaks, systemic gaps in WASH, health care infrastructure, and public health preparedness persist. The impact of these outbreaks on lives, health care systems, and the economy demands immediate action. Zambia must prioritize investments in WASH, strengthen epidemic surveillance, and adopt innovative technologies such as predictive modeling and AI to enhance preparedness. Community engagement, public health education, and addressing social stigma are critical for behavior change and early case reporting.

This write-up paves way for future studies that will investigate how AI and mathematical modeling can be integrated into cholera prediction systems while evaluating the quality of available data and identifying gaps in information technology (IT) infrastructure that may hinder implementation. It would also be prudent to explore cost-effective strategies to improve real-time data collection in high-risk areas to strengthen outbreak prediction and early response mechanisms.

## Declarations of competing interest

The author have no competing interests to declare.
